# m^5^C RNA Methylation Primarily Affects the ErbB and PI3K–Akt Signaling Pathways in Gastrointestinal Cancer

**DOI:** 10.3389/fmolb.2020.599340

**Published:** 2020-12-07

**Authors:** Shixin Xiang, Yongshun Ma, Jing Shen, Yueshui Zhao, Xu Wu, Mingxing Li, Xiao Yang, Parham Jabbarzadeh Kaboli, Fukuan Du, Huijiao Ji, Yuan Zheng, Xiang Li, Jing Li, Qinglian Wen, Zhangang Xiao

**Affiliations:** ^1^Laboratory of Molecular Pharmacology, Department of Pharmacology, School of Pharmacy, Southwest Medical University, Luzhou, China; ^2^South Sichuan Institute of Translational Medicine, Luzhou, China; ^3^Neijiang Health and Health Vocational College, Neijiang, China; ^4^Department of Oncology and Hematology, Hospital (T.C.M.) Affiliated to Southwest Medical University, Luzhou, China; ^5^Department of Oncology, Affiliated Hospital of Southwest Medical University, Luzhou, China

**Keywords:** m^5^C, RNA methylation, ErbB, PI3K–Akt, gastrointestinal cancer, survival

## Abstract

5-Methylcytosine (m^5^C) is a kind of methylation modification that occurs in both DNA and RNA and is present in the highly abundant tRNA and rRNA. It has an important impact on various human diseases including cancer. The function of m^5^C is modulated by regulatory proteins, including methyltransferases (writers) and special binding proteins (readers). This study aims at comprehensive study of the m^5^C RNA methylation-related genes and the main pathways under m^5^C RNA methylation in gastrointestinal (GI) cancer. Our result showed that the expression of m^5^C writers and reader was mostly up-regulated in GI cancer. The *NSUN2* gene has the highest proportion of mutations found in GI cancer. Importantly, in liver cancer, higher expression of almost all m^5^C regulators was significantly associated with lower patient survival rate. In addition, the expression level of m^5^C-related genes is significantly different at various pathological stages. Finally, we have found through bioinformatics analysis that m^5^C regulatory proteins are closely related to the ErbB/PI3K–Akt signaling pathway and *GSK3B* was an important target for m^5^C regulators. Besides, the compound termed streptozotocin may be a key candidate drug targeting on GSK3B for molecular targeted therapy in GI cancer.

## Introduction

Gastrointestinal (GI) cancer is one of the leading causes of death worldwide. It refers to cancers of the upper and lower digestive tracts and mainly includes colorectal adenocarcinoma (CRC), gastric cancer (GC), pancreatic cancer (PC), hepatocellular carcinoma (HCC), and esophageal cancer (EC) (Toomey et al., 2013). Nearly 4.1 million people are diagnosed with GI cancer each year. Epigenetic changes are common events in both initiation and progression of GI cancer (Vedeld et al., 2018). Currently, there are many ways to treat GI cancer. However, most of the treatment outcomes are still poor (Bilgin et al., 2017).

RNA methylation modifications mainly include m^6^A, m^5^C, m^1^A, m^7^G, and so on. Previous studies have shown that these modifications play important roles in the stability, processing, and genetic information transmission of mRNA (Liu and Jia, 2014; Oerum et al., 2017). Known mutations in RNA-modifying enzymes are closely related to human diseases, including cancers, cardiovascular diseases, metabolic diseases, and mitochondrial-related defects (Jonkhout et al., 2017). The degree of methylation of specific genes can be used as a diagnostic indicator of cancer (Li W. et al., 2017; Traube and Carell, 2017). 5-Methylcytosine (m^5^C) includes DNA and RNA methylation modifications, in which the methyl group is transferred to a specific base by using *S*-adenosylmethionine (SAM) as a methyl donor under the catalysis of methyltransferase. m^5^C RNA modification has been found to be highly abundant in tRNA and rRNA (Motorin et al., 2010). Meanwhile, high throughput sequencing has been used to verify the widespread presence of m^5^C in non-coding RNA and coding RNA (Squires et al., 2012). It has been reported that m^5^C modification controls many functions: protein translational regulation, RNA processing, regulating stem cell function and stress response, and promoting tRNA stability and protein synthesis (Aslan et al., 1967; Blanco et al., 2016; Liu et al., 2017; Tuorto et al., 2012). However, the involvement of m^5^C modification in GI cancer has not been systematically reported yet.

5-Methylcytosine modification is regulated by methyltransferases (writers, including *NSUN1-7* and *TRDMT1* [*tRNA aspartic acid methyltransferase 1*]) and binding protein (reader, i.e., *ALYREF* [*Aly/REF export factor*]). *NSUN1-7* and *TRDMT1* are known writers for chemical RNA modifications (Jacob et al., 2017). *NSUN1* (NOP2 nucleolar protein/rRNA MTase) plays an important role in maintaining cell proliferation capacity and is possibly involved in the regeneration of nervous tissue (Kosi et al., 2015; Hong et al., 2016). *NSUN2* (*NOP2/Sun RNA methyltransferase 2/*mRNA and tRNA MTase) is a main RNA modification methyltransferase. Its mechanism of action includes controlling cell division, growth arrest, and promoting premature senescence (Xing et al., 2015; Cai et al., 2016; W. Wang, 2016; Yang X. et al., 2017). It has been reported that *NSUN2* mutations lead to intellectual disability in human diseases (Abbasi-Moheb et al., 2012). *NSUN3* (NOP2/Sun RNA methyltransferase 3/mt-tRNA MTase) and *NSUN4* (NOP2/Sun RNA methyltransferase 4/mt-rRNA MTase) have important impacts on the mitochondria (Metodiev et al., 2014; Schosserer et al., 2015; Nakano et al., 2016). *NSUN5* (NOP2/Sun RNA methyltransferase 5/rRNA MTase) is a conserved rRNA methyltransferase (Schosserer et al., 2015). *NSUN6* (NOP2/Sun RNA methyltransferase 6/tRNA MTase) modifies tRNAs in their biogenesis (Haag et al., 2015). *NSUN7* (*NOP2/Sun RNA methyltransferase family member 7*) gene product plays a role in sperm motility (Khosronezhad et al., 2015). *TRDMT1* (also known as *DNMT2*) was previously considered as a DNA MTase, but it is now primarily regarded as a tRNA MTase (Schaefer and Lyko, 2010; Squires et al., 2012; Jeltsch et al., 2017). Up to now, the m^5^C eraser is still unknown, and the only known binding protein (reader) of m^5^C is *ALYREF*. *ALYREF* as an m^5^C reader can promote mRNA export (Yang X. et al., 2017). In general, m^5^C methyltransferases are strongly associated with diseases.

Currently, there is little research progress in the biological function and mechanism of m^5^C in GI cancer. In this study, we analyzed the gene expression level, alteration frequency, and association with survival of m^5^C regulators in GI cancer. Meanwhile, we also studied their related pathways and key target, for which a druggable compound was found in the hope of providing new treatment for patients with GI cancer.

## Materials and Methods

### Data Processing

The expression level and clinical data of m^5^C regulators in five types of GI cancers were extracted from the TCGA database^1^ (Tomczak et al., 2015) (download date: 2019-05-05). There were 1,696 cancer samples and 148 normal samples. The standardized TCGA and GTEX transcriptome data are derived from the UCSC database^2^. In total, there were 1,451 cancer samples and 1,044 normal samples.

### Somatic Alteration Analysis

The cBioportal analysis of the GISTIC 2.0 database was used to analyze the alteration frequency and percentage of m^5^C regulatory factors in GI cancers and protein affected by m^5^C regulators (Cerami et al., 2012). OncoPrint can summarize distinct genomic alterations across samples in the m^5^C regulatory factors, including mutations, CNAs, and changes in gene expression or protein abundance (Gao et al., 2013).

### Protein Structure Alteration

The protein structure alteration was analyzed in cBioportal using the Mutations tab. The query was limited to respective m^5^C regulatory factors in different types of GI cancer. Lollipop of each protein structure change of GI cancer was linked to COSMIC (Tate et al., 2019). The detailed mutation annotation was originated from OncoKB, CIViC, and Hotspot (Tate et al., 2019).

### Pathway Analysis

Proteomic data were collected by Reverse Phase Protein Array (RPPA) based on TCGA data from cBioportal^3^. For the enriched proteins, significant change in expression was determined by the standard of log_2_ based ratio (μ mean altered/μ mean unaltered) (log > 0 for over-expression and log < 0 for under-expression) and queried event results in *P* value <0.05. The -log_10_ *P* value >1.30 proteins were selected for further downstream pathway analysis. Differential proteins are shown by the volcano plot using GraphPad Prism 7. The selected proteins from this criterion were used to predict pathways by two conditions: (a) the sum of altered protein in each pathway and (b) the statistical *P* value score of significant pathway (Li D. et al., 2020). Finally, the screened differential proteins were used to predict the pathway in the DAVID function annotation tool^4^.

### Gene Ontology Analysis

The Gene Ontology (GO) enrichment analysis of the m^5^C RNA methylation modification was analyzed *via* the DAVID function annotation tool (Dennis et al., 2003). GO contains biological processes, cell components, and molecular functions. In this analysis, count represents the number of genes contained in the GO term. Therefore, the count and *P* values are considered together to obtain important metabolic process.

### Protein–Protein Interaction Network Analysis

We analyzed the network of interactions between proteins by using the STRING and Cytoscape software. The STRING database is a meta resource, including both physical and functional interactions (Jensen et al., 2009). STRING can be reached at http://string-db.org/. The minimum required interaction score is set to medium confidence and select all active interaction sources. Cytoscape is a public software that can integrate models of biomolecular interaction networks (Shannon et al., 2003).

### Correlation and Co-expression Analysis

To better understand the co-expression between m^5^C regulatory factors and the differentially expressed genes (DEGs) associated with key downstream pathways, we used the R statistical software by R package heatmap (Chan, 2018). Parameters of co-expression analysis were set as: 0.8–1.0 strongly correlated, 0.6–0.8 strong correlation, 0.4–0.6 moderate intensity correlation, 0.2–0.4 weak correlation, and 0.0–0.2 very weak correlation or no correlation. Correlation between *GSK3B* (*glycogen synthase kinase 3 beta*) and m^5^C regulators was analyzed using the linear regression. The 95% confidence intervals were presented by dot lines. The data have been standardized.

### Network Pharmacology Analysis

Differentially expressed genes related to the *GSK3B* gene were obtained by R package limma. The samples were divided into two groups according to the median expression values of the *GSK3B* gene and | log_2_ fold-change (FC)| > 1, and the *P* value <0.05 was set as a threshold. According to the *P* value ranking, the first 150 DEGs that were significantly up-regulated and the first 150 DEGs that were significantly down-regulated were included for potential drug target analysis. The Connectivity Map (CMap) is a gene expression profile database based on interventional gene expression (Subramanian et al., 2017). It is mainly used to analyze the functional connections between small molecule compounds, genes, and diseases (Lamb et al., 2006). PharmMapper is a comprehensive target pharmacophore database that can search potential drug target identification (Wang et al., 2017). PharmMapper comes from TargetBank, DrugBank, BindingDB, and potential drug target databases, and nearly 53,000 receptor-based pharmacophore models are used for prediction (Liu et al., 2010; Wang et al., 2016).

### Statistical Analysis

*T* test is used for comparison between two groups of data, and one-way ANOVA is applied to compare multiple groups. Survival analysis was performed using Kaplan–Meier curve with *P* value calculated using the log-rank test. The correlation of mRNA expression was analyzed by Pearson test. Chi-square test was used to test the association of m^5^C regulator expression with clinicopathological parameters. *P* < 0.05 was considered statistically significant.

## Results

### The Expression Level of m^5^C Regulators in GI Cancer

**FIGURE 1:**
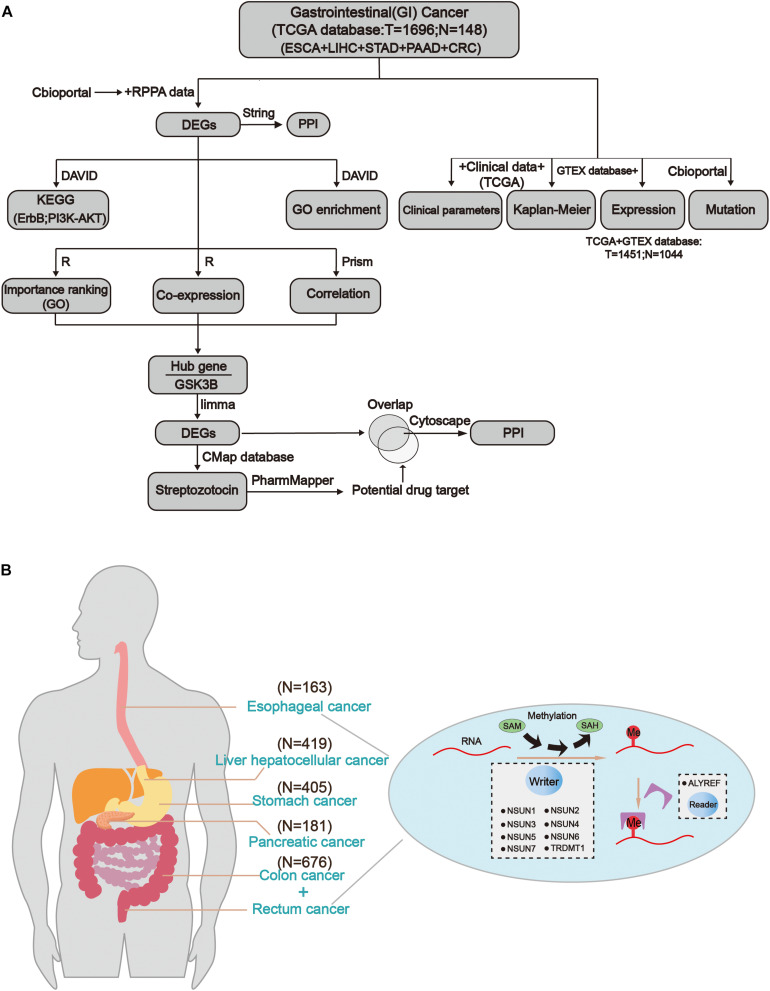
Comprehensive molecular profiling of m^5^C writers and reader in GI cancer. **(A)** The workflow of the study. **(B)** The regulation mechanism of m^5^C in GI cancers (esophageal cancer, liver cancer, gastric cancer, colon cancer, and rectal cancer). m^5^C formation is catalyzed by writer and SAM (*S*-adenosylmethionine). In addition, reader can recognize methylated mRNA and mediate its export from the nucleus. They work together on m^5^C RNA methylation modification.

**FIGURE 2:**
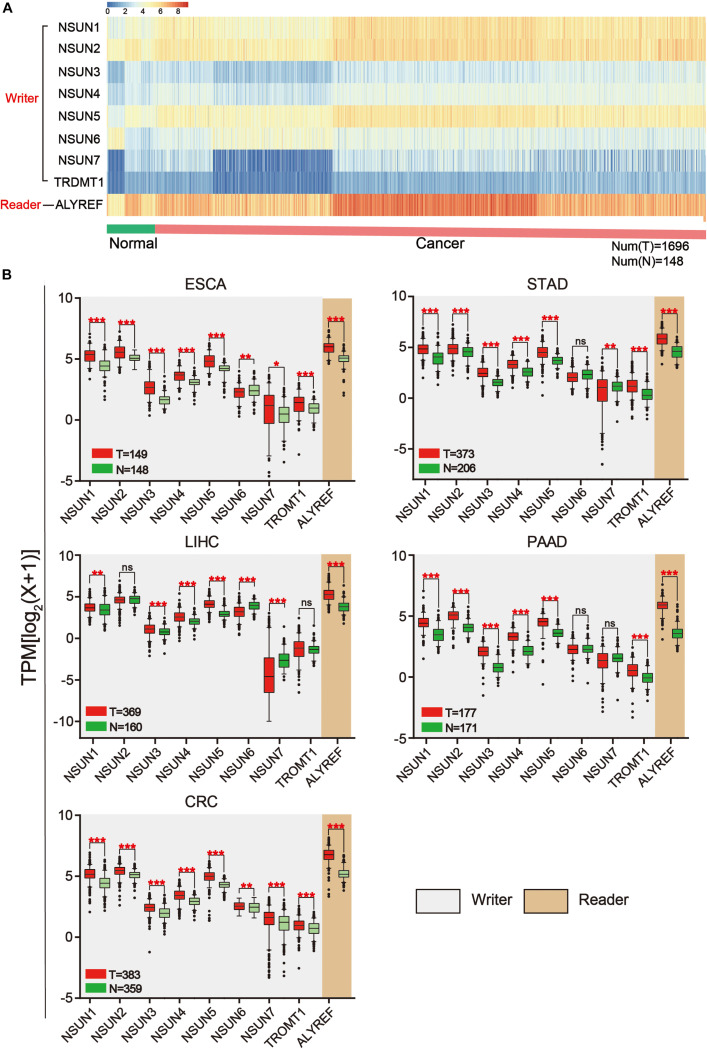
Expression of m^5^C writers and reader in GI cancer. **(A)** Based on RNA sequencing data from the TCGA database, a heat map of m^5^C writer and reader expression was drawn. Data were collected from cancer patients (*n* = 1,568) and healthy patients (*n* = 139). Each sample was normalized and was represented by a square. Red and blue regions represented higher and lower expression levels, respectively. **(B)** RNA sequencing data were used to compare the expression level of each m^5^C regulator between tumor and normal samples in different GI cancer types.

The workflow of the study and nomenclature and mechanism of m^5^C writer and reader was demonstrated in Figures 1A,B. To characterize the expression of m^5^C writers and reader in GI cancer, we first used the TCGA data. Overall, the expression level of *NSUN3*, *NSUN4*, *NUSN6*, *NUSN7*, and *TRDMT1* was lower than that of other m^5^C regulators (Figure 2A). When comparing the expression level between 1,696 cancer and 148 normal samples, heat map showed that the expression of m^5^C writers and reader was generally higher in GI cancer than in normal samples (Figure 2A). The combination of the TCGA and GTEX databases was used to compare the expression level of m^5^C writer and reader between tumor tissue and normal samples in GI cancer. The results indicated that writers and reader were mostly up-regulated in GI cancer (Figure 2B). Meanwhile, we did principal component analysis of gene expression in 1,695 samples of the five cancer types (Supplementary Figure S1). X and Y axes explained 39.5 and 16.4% of the total variance, respectively. The further apart the two samples, the greater the difference in genetic background between them would be. From the figure, the five cancers were almost distinctively separated.

### Mutation of m^5^C Regulators

**FIGURE 3:**
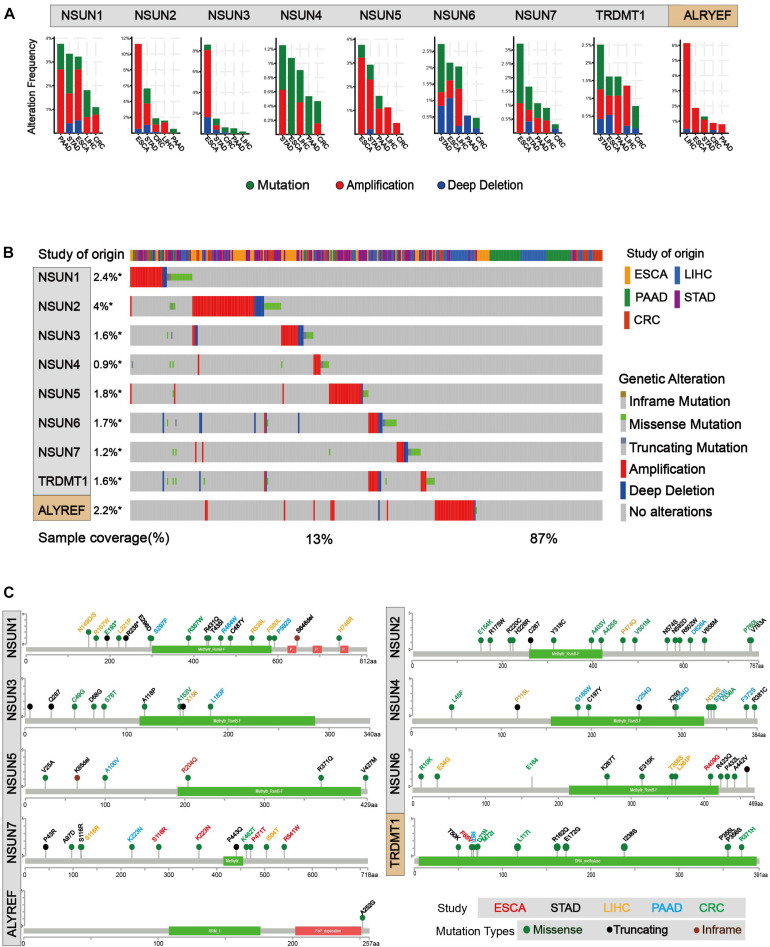
Mutations of m^5^C writer and reader in GI cancer. **(A)** Alteration frequency of m^5^C regulatory factors in GI cancer, including mutations, amplification, and deletion, and multiple changes were analyzed from the TCGA database and studied in cBioPortal. About 28% (52/186) of esophageal cancer, 8% (14/186) of pancreatic cancer, 19% (91/478) of stomach cancer, 5% (33/640) of colorectal cancer, 13% (56/442) of liver cancer, and 12% (234/1,928) of GI cancer had genomic changes of m^5^C regulators. **(B)** The alteration frequency of m^5^C regulatory factors in gastrointestinal cancer from the TCGA database was analyzed in cBioPortal. The gene alteration frequencies of m^5^C regulators in GI cancer was 2.4% in *NSUN1*, 4% in *NSUN2*, 1.8% in *NSUN5*, and 2.2% in *ALYREF*, etc. **(C)** Protein structure alteration (missense, truncating, and inframe mutation) was analyzed in GI cancers.

In order to identify mutations of m^5^C regulators, cBioPortal for Cancer Genomic was used (Cerami et al., 2012; Gao et al., 2013). As shown in Figures 3A,B, mutation and amplification were frequently seen in m^5^C regulators. *NSUN2*, *NSUN5*, and *ALYREF* showed relatively higher copy number amplification. In Figure 3B, we also found that there are many types of mutations in m^5^C RNA methylation regulators, such as inframe mutation, missense mutation, amplification, and deep deletion. Among them, amplification has the largest proportion of types, and deep deletion also accounts for a large proportion. Overall, about 13% of the samples (246/1,924) had genetic changes, and the mutation rate of m^5^C regulators was relatively higher in esophagus and stomach cancers than in other cancer types. Furthermore, 28% (52/186) of EC, 8% (14/186) of PC, 19% (91/478) of stomach cancer, 5% (33/640) of colorectal cancer, 13% (56/442) of liver cancer, and 12% (234/1,928) of GI cancer had genomic changes of m^5^C regulators. Notably, the mutation frequencies of *NSUN2* in esophagus and stomach cancers, *NSUN3* in esophagus cancer, and *ALYREF* in liver cancer were particularly high. Changes in protein structure were shown in Figure 3C. Mutation in protein sequence was more often found in *NSUN1* and *NSUN2* than in other genes.

### Impact of m^5^C Regulator Alterations on Patient Survival

**FIGURE 4:**
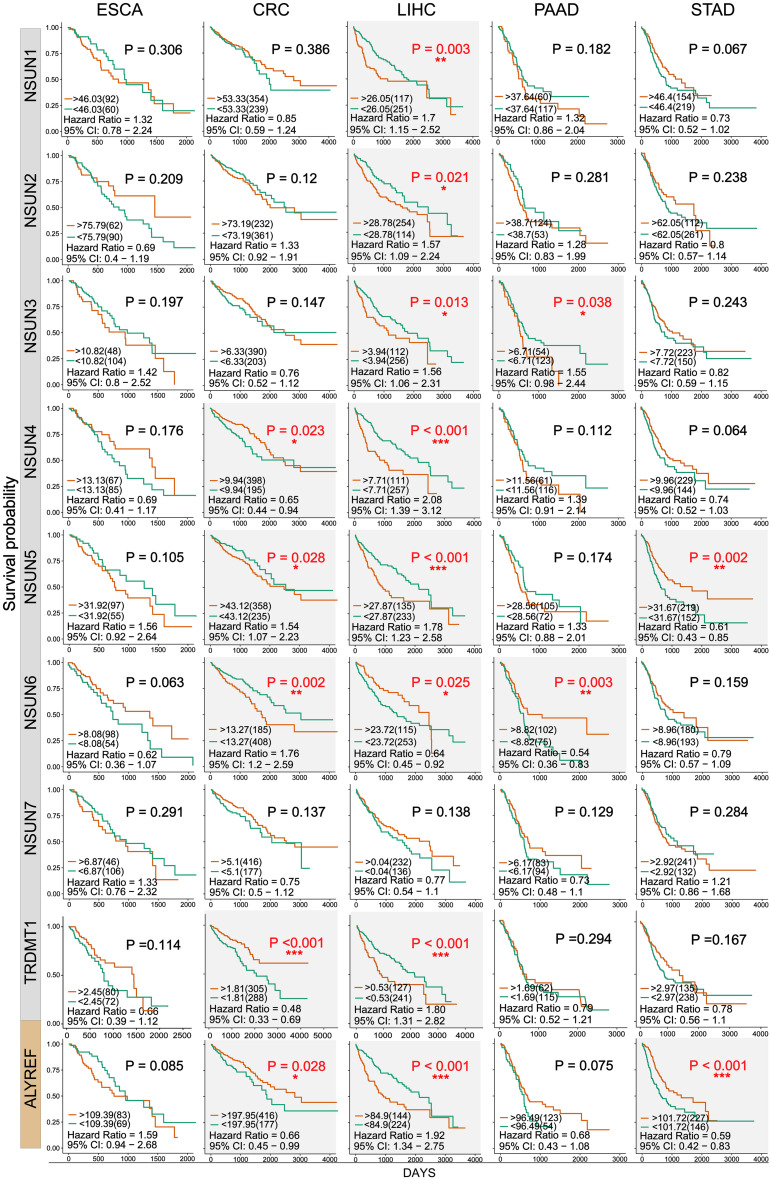
Kaplan–Meier survival curves of m^5^C regulatory factors in GI cancer. Kaplan–Meier survival curves were drawn based on the differential expression level of m^5^C regulatory factors in GI cancer. The red and green curves showed survival curves of the high and low expression groups, respectively. ^∗^*P* < 0.05, ^∗∗^*P* < 0.01, and ^∗∗∗^*P* < 0.001 between the two groups.

Next, we used clinical information in the TCGA database to evaluate the influence of m^5^C writers and reader expression on the survival rate in patients with GI cancer. Kaplan–Meier analysis showed that the differential expression of some m^5^C writers and reader was significantly related to overall survival (OS) (Figure 4 and Supplementary Table S1). In this picture, we can find that except the *NSUN7* gene, the survival rates of other genes have significant differences in the different GI cancers. Among them, the *P* value of the *NSUN5* and *ALYREF* genes in colorectal cancer, liver cancer, and GC is less than 0.05, and the *NSUN6* gene also has significant differences in colorectal cancer, liver cancer, and PC. Remarkably, high expression of almost all m^5^C regulators was significantly associated with shorter OS in HCC patients. These results suggest that m^5^C regulators may play an important role in the survival of HCC patients.

### Association of m^5^C Regulators With Clinicopathological Parameters

**FIGURE 5:**
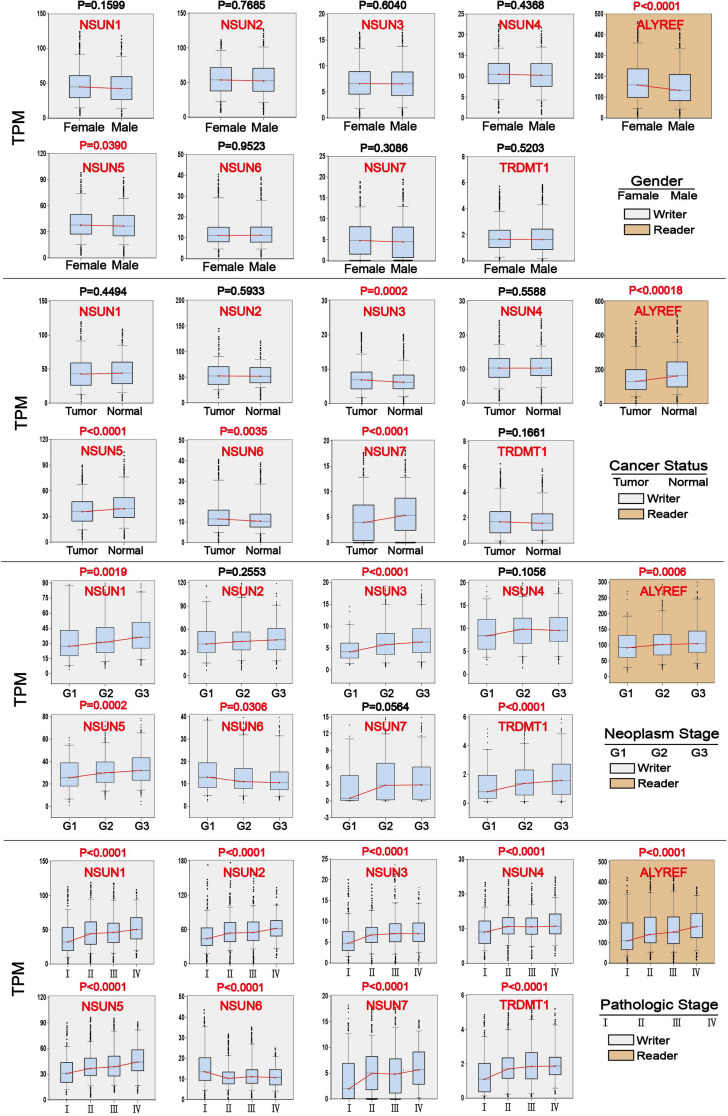
Association of m^5^C regulator expression with clinicopathological parameters in GI cancer. Clinical parameter analysis includes stage of tumor differentiation (G1, G2, and G3), pathological stage, and cancer status, and gender was analyzed by one-way ANOVA.

We investigated the association of m^5^C regulator expression with gender (male and female), cancer status (tumor and normal), tumor grade (G1, G2, and G3), and pathological stage (stage I, stage II, stage III, and stage IV) as shown in Table 1. The results showed that the overall expression of m^5^C writers was significantly associated with pathological stage and tumor differentiation grade and the expression of m^5^C reader was significantly associated with gender, cancer status, and pathological stage. The association of respective m^5^C regulator with these parameters is shown in Figure 5. The expression of *ALYREF* and *NSUN6* was significantly higher in female than in male patients. The level of *NSUN3* and *NSUN6* was increased in tumor samples, whereas the level of *ALYREF*, *NSUN5*, and *NSUN7* was decreased in tumor samples. For tumor grade, all m^5^C regulators gradually increased from G1 to G3 except *NSUN6*, which showed an opposite trend of expression. Similar to the result for tumor grade, the expression of all m^5^C regulators was elevated from pathological stage I to IV, except for *NSUN6* whose expression was lowered. We also performed the same analysis of the nine m^5^C writers and readers in different types of GI cancer and liver cancer separately (Supplementary Table S2).

**TABLE 1:** Association of m^5^C mRNA expression with clinicopathological parameters of GI cancer patients.

		Writer				Reader			
		High	Low	χ ^2^	*P*	High	Low	χ ^2^	*P*
Gender	Male	999	50	0.1591	0.6900	487	562	13.86	**0.0002**^∗∗∗^
	Female	616	28			359	285		
Cancer status	Tumor	842	48	3.321	0.0684	397	493	26.22	**<0.0001**^∗∗∗^
	Normal	573	20			345	248		
Grade	G1	103	6			47	62		
	G2	438	14	22.57	0.4798	226	226	2.651	0.4147
	G3	497	19			256	260		
Pathological stage	Stage I	334	33	8.508	**0.0366**^∗^	101	266	20.8	**0.0001**^∗∗∗^
	Stage II	580	63			233	410		
	Stage III	435	26			175	286		
	Stage IV	133	7			67	73		

*P < 0.05, **P < 0.01, and ***P < 0.001.

### Pathways Associated With m^5^C Regulators

**FIGURE 6:**
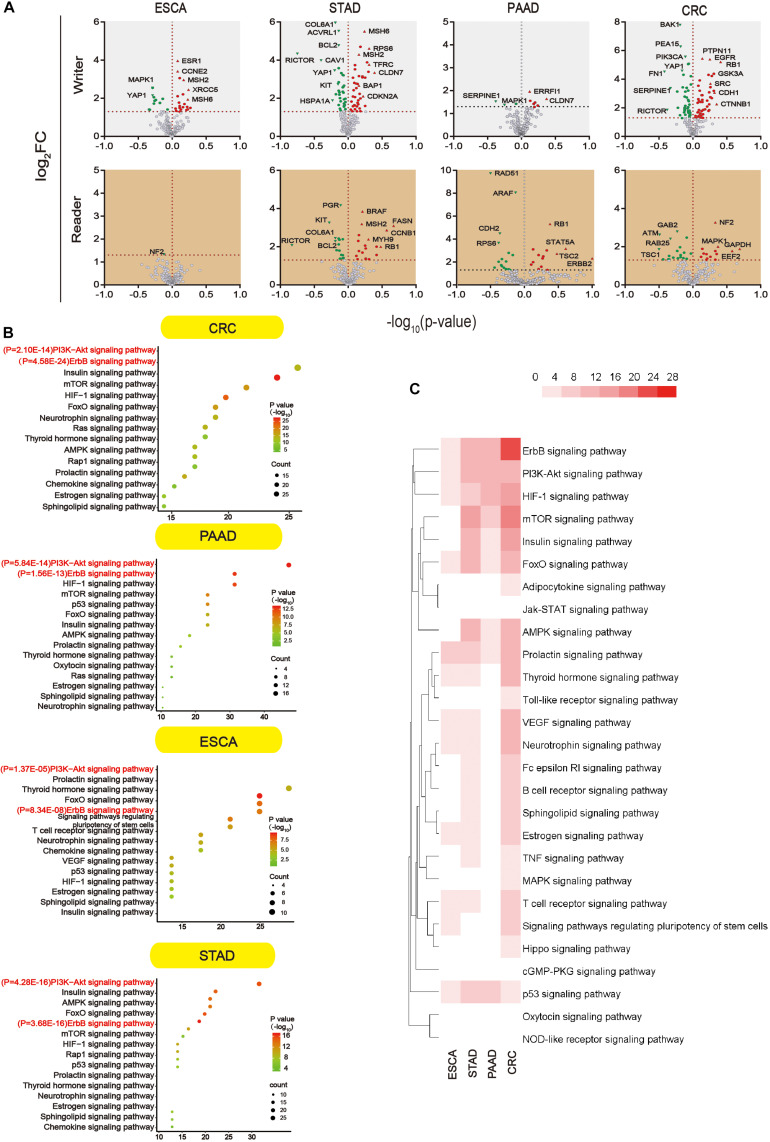
Main signaling pathways affected by m^5^C. **(A)** Volcano plot showing the proteins significantly affected by m^5^C regulators mutation in GI cancer. -log_10_ (*P* value) >1.30 was considered a significant change. **(B)** Bubble plots showing the downstream pathways of m^5^C based on gene count and *P* value. Prediction of the downstream pathways related to m^5^C gene alterations was analyzed by KEGG pathway analysis *via* DAVID. **(C)** Heat map showing the important downstream pathways of m^5^C in GI cancers based on *P* value.

We further analyzed proteins that were altered upon m^5^C regulator mutation (mutation, amplification, and deep deletion). Since data were not found in liver cancer, subsequent analysis was done in other four types of GI cancer. Proteins that showed significant changes (*P* < 0.05) were shown in the volcano map (Figure 6A). All the differential proteins were summarized in Supplementary Table S3. In order to know which pathway the differential proteins are enriched, we used DAVID for Kyoto Encyclopedia of Genes and Genomes (KEGG) proteins enrichment. According to the downstream gene count and *P* value, bubble plots were constructed in different types of GI cancer (Figure 6B). The *P* value and the number of genes are shown in this figure. More detailed data can be found in Supplementary Tables S3 and S4. In addition, a heat map was constructed (Figure 6C) considering P values of different pathways. Among 34 pathways, we found some major pathways affected by m^5^C regulators: ErbB, PI3K–Akt, HIF-1, and mTOR signaling pathways. Combining the bubble plots and heat map results, we conclude that the ErbB signaling pathway and PI3K–Akt signaling pathway are the most important downstream pathways of m^5^C RNA methylation modification.

### Function and Interaction of Downstream Pathway Proteins

**FIGURE 7:**
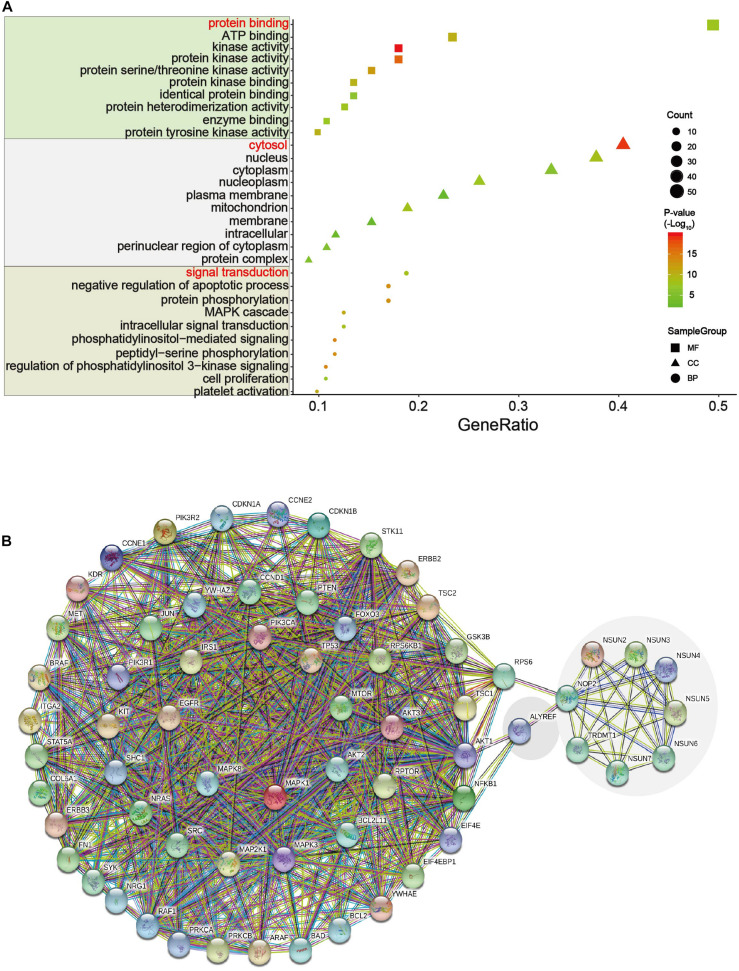
GO and network analysis of proteins in two important pathways. **(A)** Gene Ontology (GO) enrichment analysis of the m^5^C RNA methylation modification was analyzed *via* DAVID. GO contains biological processes, cell components, and molecular functions. In this picture, count represents the number of genes contained in the GO term. The count and *P* values were considered together to obtain important metabolic process. Three important metabolic processes are affected by m^5^C RNA methylation, including protein binding, cytosol, and signal transduction. **(B)** Multicenter protein–protein interaction (PPI) network between m^5^C regulatory proteins and differential proteins in the ErbB and PI3K–Akt pathways in the STRING database.

We analyzed the 54 differential proteins (Supplementary Table S3) that were enriched in the ErbB and PI3K–Akt signaling pathways by GO terms biological process enriched *via* DAVID. The result demonstrated that these proteins were mainly involved in the regulation of protein binding, cytosol, and signal transduction (Figure 7A). To further understand the interaction between these differential proteins and m^5^C regulators, we mapped protein–protein interaction (PPI) networks. The results showed that *ALYREF* and *RPS6* were important connections between differential proteins and m^5^C regulators (Figure 7B).

### *GSK3B* Is Closely Related to m^5^C Regulators

**FIGURE 8:**
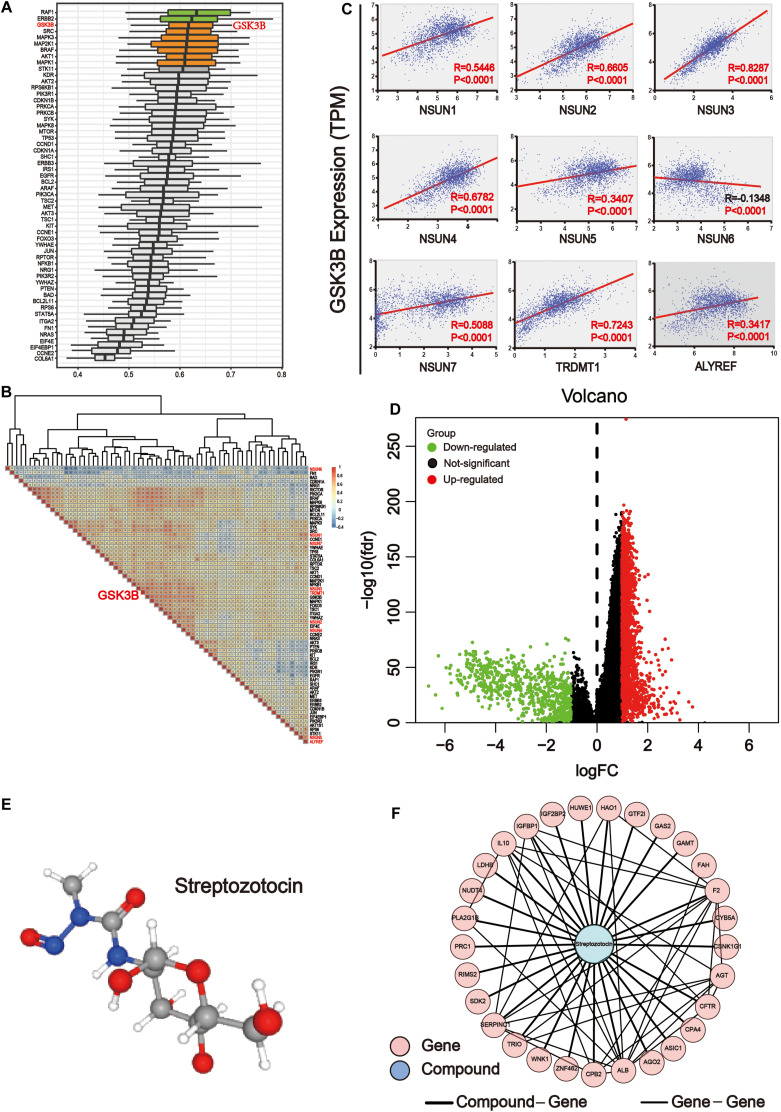
GSK3B is closely related to m^5^C regulators. **(A)** The importance of key proteins of the downstream pathway in the three major categories of Gene Ontology (GO), including biological processes, cell components, and molecular functions. The *X* axis is the score obtained by comprehensively considering the weight of the three categories of GO. The higher the score, the more important the protein is involved in the GO project. **(B)** Co-expression analysis of m^5^C differential proteins of the ErbB and PI3K–Akt pathways. The co-expression scores of related genes were displayed. The color intensity reflects the reliability of co-expression; meanwhile, red and blue indicate high correlation and low correlation, respectively. **(C)** Correlation analysis between writers and reader protein of m^5^C with *GSK3B*. There were significant differences between GSK3B and all genes. **(D)** The samples were grouped according to the median expression of GSK3B gene, and differential expression analysis was performed. **(E)** 3D structure of streptozotocin. **(F)** PPI network analysis between the common genes between streptozotocin.

In order to determine the importance of functional annotations between different proteins, we conducted analysis by using the R/Bioconductor package GOSemSim. According to the ranking results of importance, the results showed that among downstream pathway-associated proteins, *GSK3B* plays a crucial role in the three major categories of GO, including biological processes, cell components, and molecular functions. Other important proteins included AKT1S1, RAF1, ERBB2, SRC, MAPK3, MAP2K1, BRAF, AKT1, and MAPK1 (Figure 8A). Next, we used the expression level of these genes to map the correlation between them. The results showed that *GSK3B* was positively correlated with most genes (Figure 8B). Then, we analyzed the correlation of GSK3B with m^5^C regulators (Figure 8C). The result showed that *GSK3B* was significantly and positively correlated with all m^5^C regulators except for *NSUN6* for which it showed negative correlation, indicating that *GSK3B* protein may be closely related to m^5^C writer and reader. In the human disease methylation database (DiseaseMeth version 2.0), we found that *GSK3B* is not influenced by DNA methylation in GI cancer (Supplementary Figure S2). In conclusion, we believe that m^5^C writer and reader mainly affect genes in the ErbB/PI3K–Akt signaling pathway, and that *GSK3B* may be an important downstream target of m^5^C regulators.

### Network Pharmacology Analysis of the *GSK3B* Gene in GI Cancer

The transcriptome data of GI cancer were integrated, and tumor samples were divided into the high and low expression groups according to the median level of the *GSK3B* gene expression. In total, 2,071 significantly up-regulated and 695 significantly down-regulated genes were identified (Figure 8D). In order to find the probable drug targeting the *GSK3B* gene *via* the CMap database, we screened the first 150 genes in the up-regulated and down-regulated genes, respectively. Streptozotocin was the highest score in the prediction. Next, the PubChem database was applied to obtain the 3D structure of streptozotocin (Figure 8E). Next, 280 target genes were gained through the PharmMapper database. Then, we chose the common genes between 274 drug targets and 2,766 differential genes of the *GSK3B* gene, and finally, 29 genes were used for PPI network with this compound on streptozotocin, including *HUWE1*, *AGT*, *HAO1*, *CPB2*, *WNK1*, *CPA4*, *ZNF462*, *RIMS2*, *GAS2*, *CFTR*, *PLA2G1B*, *F2*, *SERPINC1*, *TRIO*, *FAH*, *CSNK1G1*, *AGO2*, *PRC1*, *ASIC1*, *CYB5A*, *GTF2I*, *IL10*, *IGFBP1*, *SDK2*, *GAMT*, *LDHB*, *ALB*, *NUDT4*, and *IGF2BP2* (Figure 8F).

## Discussion

5-Methylcytosine modifications of RNA are ubiquitous in nature and play important roles in many biological processes, such as protein translational regulation, RNA processing, and stress response (Hussain et al., 2013; Yuan et al., 2014; Liu et al., 2017); RNA stability (Tuorto et al., 2012; Zhang et al., 2012); RNA transport (Yang X. et al., 2017); and mRNA translation (Tang et al., 2015; Xing et al., 2015; Sun et al., 2019). Currently, the modification mechanism of m^5^C in cancer is being explored. Nonetheless, the exact catalytic mechanism of m^5^C methylation remained unclear (Li Q. et al., 2017; Liu et al., 2017; Trixl and Lusser, 2019). Herein, we conducted a comprehensive analysis of known m^5^C writers (*NSUN1-7* and *TRDMT1*) and reader (*ALYREF*) in GI cancer. Overall, the expression level of m^5^C regulators was distinctly different across all samples (Figure 2A). Notably, the expression of *NSUN1*, *NSUN3*, *NSUN4*, *NSUN5*, and *ALYREF* was all significantly elevated in GI cancer (Figure 2B). Meanwhile, the genomic and protein structure alterations of m^5^C regulators were also determined (Figure 3). Overall, the mutation rate of m^5^C regulators in GI cancer was not high, and it was relatively higher in esophagus and stomach cancers than in other cancers (Figures 3A,B). The mutation rate for *NSUN2* was the highest. In accordance with our finding, copy number gain of *NSUN2* has been reported in breast, oral, and colorectal cancers (Frye et al., 2010; Okamoto et al., 2012), which leads to the increased expression of it in cancers. Alteration in protein structure was seen in all m^5^C regulators (Figure 3C). There were more protein alteration sites in *NSUN1* and *NSUN2* than in other regulators, whereas there was only one alteration site in *ALYREF*. Although study on the role of m^5^C regulatory proteins in cancer was very limited, *NSUN2* was relatively well-studied among m^5^C regulators. There were a few reports on the elevated expression of *NSUN2* in a range of cancer, including oral (Okamoto et al., 2012), head and neck (Lu et al., 2018), colorectal (Okamoto et al., 2012), breast (Frye et al., 2010; Yi et al., 2017), ovarian (Yang J. C. et al., 2017), and GI cancers (Okamoto et al., 2012), which was consistent with our bioinformatics analysis. *TDMT1*/*DNMT2*, a member of DNA methyltransferases, was shown to be down-regulated in liver (Saito et al., 2001), stomach, and colorectal cancers (Kanai et al., 2001). In contrast, it was significantly over-expressed in stomach and liver cancers from our study and decreased in PC (Figure 2B).

Next, we used clinical information to evaluate the association of m^5^C regulator expression with patient survival and clinicopathological parameters. Notably, high expression of almost all m^5^C regulators was significantly associated with shorter OS in HCC patients except *NSUN7*, indicating that dysregulation of m^5^C regulators may strongly influence liver cancer patient survival (Figure 4 and Supplementary Table S1). High expression of *NSUN2* has been reported to predict poor survival in head and neck cancer (Lu et al., 2018). It was only found to be associated with shorter survival in liver cancer from our study. For clinicopathological parameters, consistent with previous result (Figure 2B), the level of *NSUN3* and *NSUN6* was increased in tumor samples versus normal samples (Figure 5). For tumor grade, the expression of m^5^C regulators increased from G1 to G3 except *NSUN6*, which showed an opposite trend of expression. The result for pathological grade was similar to that for tumor grade. The expression of all m^5^C regulators was elevated from pathological stage I to IV, except for *NSUN6* whose expression was decreased (Figure 5). Similar to our result, *NSUN2* has been found to be significantly correlated with clinical stage and pathological differentiation in breast cancer (Yi et al., 2017).

In an attempt to find out the major targets or pathways modulated by m^5^C regulators, we first determined proteins that were significantly altered upon m^5^C regulator gene alteration. The results demonstrated that alteration in m^5^C regulators led to the decreased expression of oncogenic YAP1 and RICTOR and increased expression of DNA mismatch repair proteins MSH2 and MSH6 (Figure 6A). We then further determined which signaling pathways these differential proteins mainly belong to. By taking the gene count and P value into account, we found that PI3K–Akt and ErbB were the most important pathways affected by m^5^C regulators among other pathways including the mTOR and HIF-1 pathways (Figures 6B,C). The differential proteins in the PI3K–Akt and ErbB pathways play important roles in regulating protein binding, cytosol, and signal transduction from GO analysis (Figure 7A). The PI3K–Akt and ErbB pathways are important cancer-related pathways. Studies have shown that the ErbB signaling pathway is regulated by miR-200a/141 in the epithelial–mesenchymal transition (EMT)-related microRNA-200 family in renal cell carcinoma (RCC) (Yoshino et al., 2013). At the same time, accumulating evidence has elucidated that the PI3K–Akt signaling pathway is highly activated (Guo et al., 2015; Hao et al., 2019) and is a validated therapeutic target in RCC (Lin et al., 2014). Key miRNAs and target genes have been reported to be mainly related to the PI3K–Akt signaling pathway in GI cancers (Lai et al., 2019). A bioinformatics analysis showed that the DEGs of EC compared with normal tissues are mainly enriched in the PI3K–Akt signaling pathway (Li M. et al., 2020; Yu-Jing et al., 2020). Moreover, GLI1 co-expressed and DEGs between tumor samples and normal tissues were both largely enriched in the PI3K–Akt pathway in STAD (Yu et al., 2018; Li et al., 2019). In PAAD, 4-miRNA as independent prognostic factor was found to be related to the PI3K–Akt signaling pathway (Wang et al., 2019). Growing evidence revealed that the ErbB and PI3K–Akt signaling pathways play vital roles in colorectal cancer by regulating microRNA, lncRNA, mRNA, etc. (Szmida et al., 2015; Song et al., 2018; Wei et al., 2019; Zhong et al., 2019; Wan et al., 2020). These findings indicate that GI cancer is closely related to the ErbB and PI3K–Akt signaling pathways. By visualizing the PPI network of m^5^C regulators and their potential downstream targets in the PI3K–Akt and ErbB pathways, we found that m^5^C regulators formed a group and were closely connected with the differential protein group by NOP2, ALYREF, and RPS6 (Figure 7B).

Further analysis revealed that *GSK3B* was an important potential target for m^5^C regulators (Figure 8). It showed strong association with m^5^C regulators (Figure 8B) and differential proteins and was also important in GO biological processes (Figure 8A). Importantly, *GSK3B* was significantly and positively associated with nearly all m^5^C regulators, whereas it was negatively correlated with NSUN6, indicating that it probably is a downstream target of m^5^C regulators (Figure 8C). In order to further explore the targeted drugs of the *GSK3B* gene in GI cancer, we divided the tumor samples into two groups based on the median GSK3B expression level for differential expression analysis (Figure 8D). The first 150 genes were selected from the significantly up-regulated and down-regulated differential genes, respectively, for potential drug target analysis. The results showed that streptozotocin (P-selectin inhibitor) was used for further analysis with the highest score of 96.16 (Figure 8E). Next, the targeted gene of the compound on streptozotocin was identified *via* the PharmMapper database, then we found 29 common genes of the gene target and GSK3B differential genes, and PPI network was used to display the relationship of 29 genes and the streptozotocin (Figure 8F). *GSK3B* has been shown to be frequently up-regulated in many types of cancer (Darrington et al., 2012; T. Zhang et al., 2019), and inhibition of it was considered efficient in suppressing tumor growth (Edderkaoui et al., 2018; Wu et al., 2019). Moreover, there have been many studies on GSK3B inhibitors, including Metavert molecule in PAAD (Edderkaoui et al., 2018), BT-000775 molecule in BRCA (Ogunleye et al., 2019), BIO molecule in TNBCs (triple-negative breast cancers) (Vijay et al., 2019), AR-A014418 and SB-216763 molecules in STSs (soft tissue sarcomas) (Abe et al., 2020), etc.

In summary, our study demonstrated for the first time the comprehensive analysis of m^5^C modulators in GI cancer. The dysregulation of m^5^C regulators in GI cancer was shown, its association with patient survival and clinicopathological parameters were analyzed, and the main downstream pathway and major target were determined. Besides, the compound termed streptozotocin may be a key candidate drug for targeted therapy in GI cancer. This is a pioneer study of the relationship between m^5^C dysregulation and cancer, but our results lack experimental verification, which warrants further validation of the involvement of m^5^C regulators and their downstream targets in GI cancer.

## Data Availability Statement

The original contributions presented in the study are included in the article/Supplementary Material, further inquiries can be directed to the corresponding author/s.

## Author Contributions

SX, YM, and QW analyzed the data and wrote the manuscript. JS, ZX, and QW provided funding. YSZ, XW, and ML designed the study. PK and YZ prepared and adjusted the figures. XY, XL, and JL reviewed and revised the manuscript. All authors contributed to the article and approved the submitted version.

## Conflict of Interest

The authors declare that the research was conducted in the absence of any commercial or financial relationships that could be construed as a potential conflict of interest.

## Footnotes

1 http://cancergenome.nih.gov

2 https://xenabrowser.net/

3 http://www.cbioportal.org/

4 https://david.ncifcrf.gov

## Supplementary Material

Supplementary Figure 1 PCA analysis of GI cancer. Principal component analysis of liver cancer, colorectal cancer, gastric cancer, pancreatic cancer, and esophageal cancer according to genes expression level. *N* = 1,695 data points. *X* and *Y* axes show principal component 1 and principal component 2 that explain 39.5 and 16.4% of the total variance, respectively. Prediction ellipses are such that with probability 0.95, a new observation from the same group will fall inside the ellipse. The further apart the two samples are, the greater the difference in genetic background between them will be.

Supplementary Figure 2 DNA methylation analysis of differential genes in GI cancer. DNA methylation analysis of key downstream pathways in GI cancer. The darker the blue, the lower the DNA methylation, and the darker the red, the higher the DNA methylation.

Supplementary Table 1 Survival curve data of m^5^C writer and reader in GI cancer.

Supplementary Table 2 The data of Chi-square analysis on cancer status (tumor and normal), pathological stage (stage I, stage II, stage III, and stage IV), gender (male and female), and tumor differentiation (G1, G2, and G3) in different types of GI cancer and liver cancer separately.

Supplementary Table 3 Differential proteins in important pathways.

Supplementary Table 4 The data of different pathways in GI cancer.
