# Supplementary figures and images for: m5C RNA Methylation Primarily Affects the ErbB and PI3K–Akt Signaling Pathways in Gastrointestinal Cancer
Source: Front Mol Biosci. 2020 Dec 7;7:599340. doi: 10.3389/fmolb.2020.599340 (PMC7750483; doi:10.3389/fmolb.2020.599340)

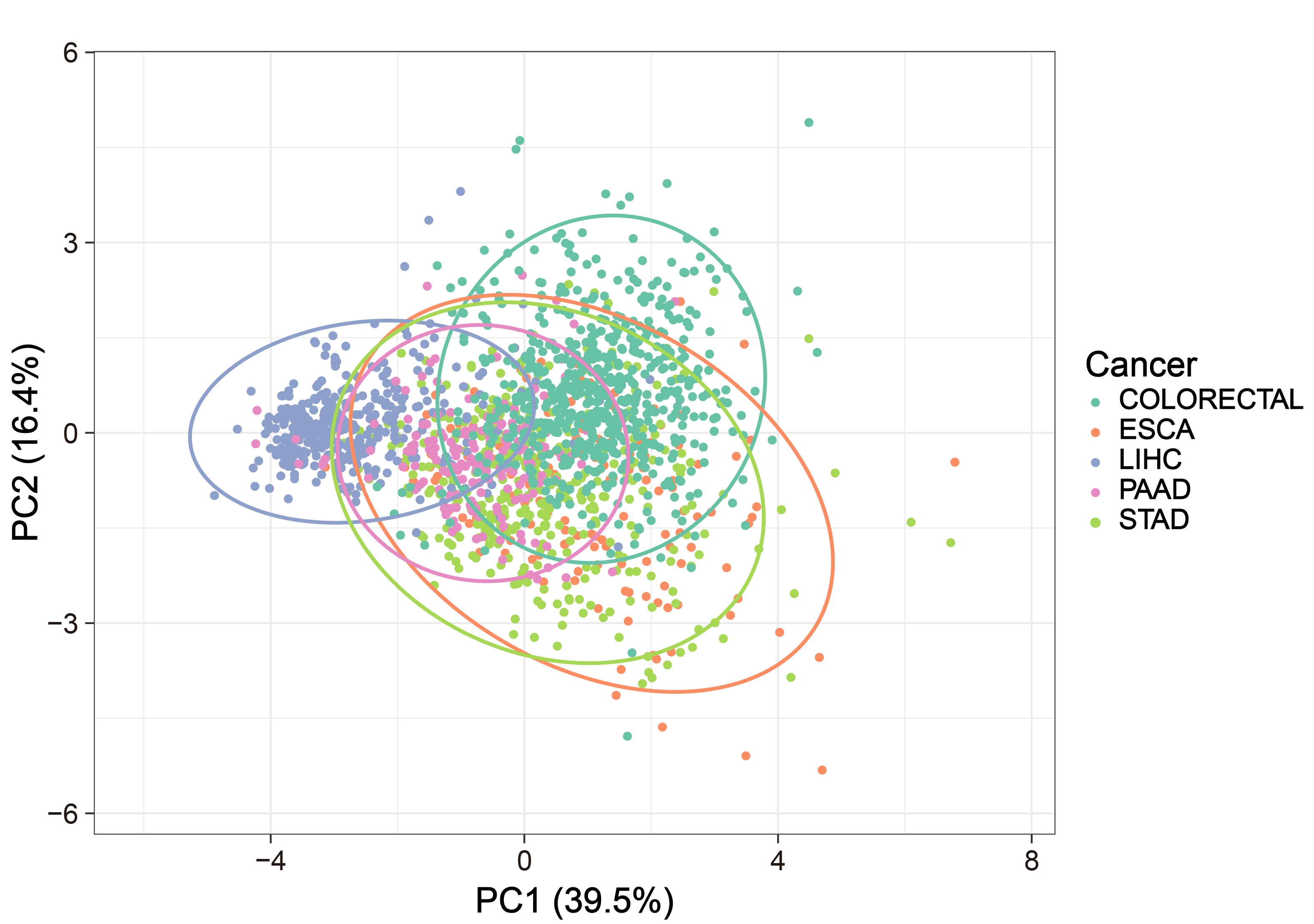

Supplement: Supplementary Figure 1 — PCA analysis of GI cancer. Principal component analysis of liver cancer, colorectal cancer, gastric cancer, pancreatic cancer, and esophageal cancer according to genes expression level. N = 1,695 data points. X and Y axes show principal component 1 and principal component 2 that explain 39.5 and 16.4% of the total variance, respectively. Prediction ellipses are such that with probability 0.95, a new observation from the same group will fall inside the ellipse. The further apart the two samples are, the greater the difference in genetic background between them will be. [file Image_1.JPEG]

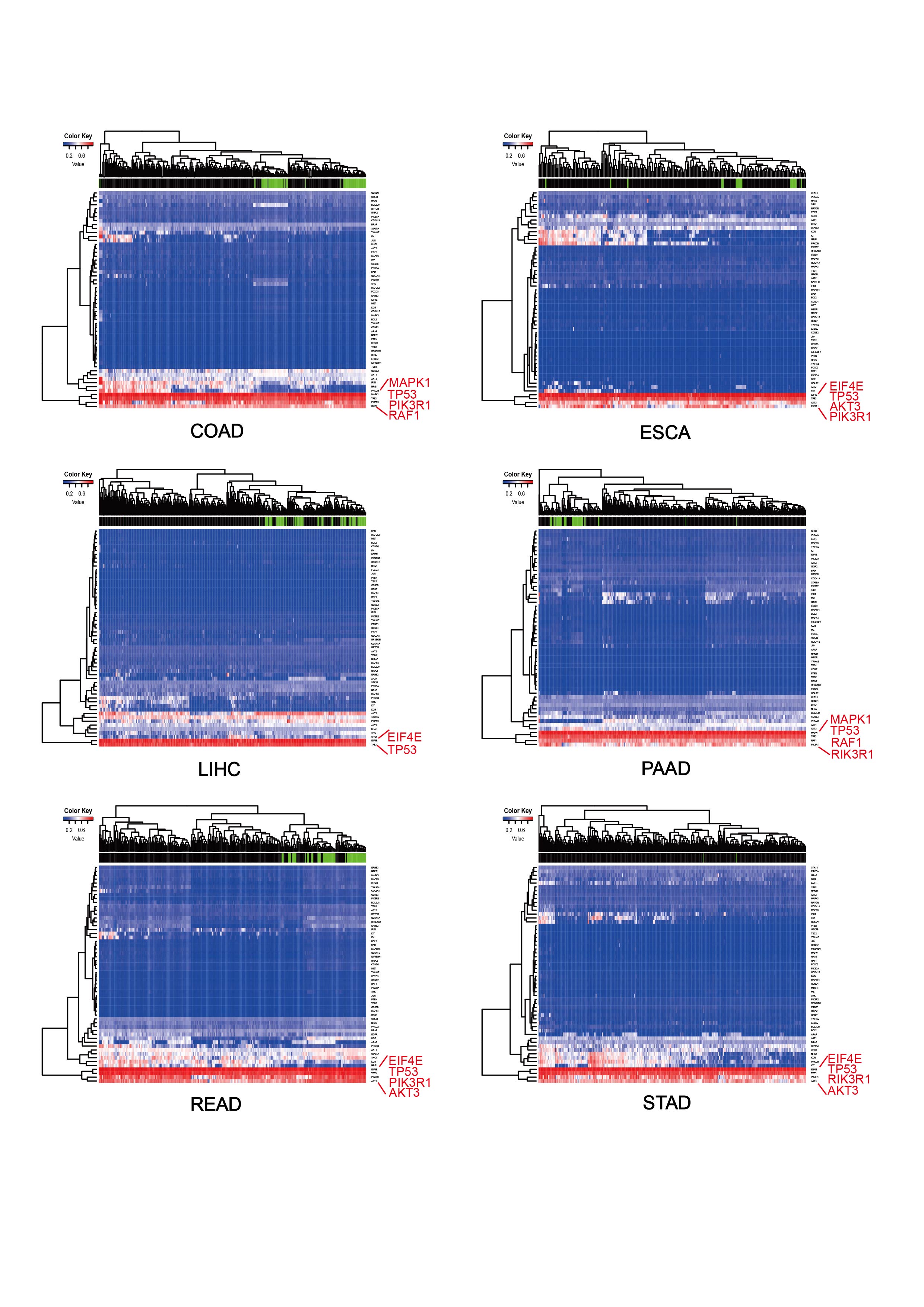

Supplement: Supplementary Figure 2 — DNA methylation analysis of differential genes in GI cancer. DNA methylation analysis of key downstream pathways in GI cancer. The darker the blue, the lower the DNA methylation, and the darker the red, the higher the DNA methylation. [file Image_2.JPEG]
